# Supplementary material for: The demand for online grocery shopping: COVID-induced changes in grocery shopping behavior of Canadian consumers
Source: PLoS One. 2024 Feb 8;19(2):e0295538. doi: 10.1371/journal.pone.0295538 (PMC10852330; doi:10.1371/journal.pone.0295538)
Supplement: S1 File — (DOCX) [file pone.0295538.s001.docx]

**APPENDIX 1**

DETAILED SUMMARY OF THE STUDY VARIABLES

| Variable | Survey question and variable description |
| --- | --- |
| **DEPENDENT VARIABLE** |  |
| *Model 1*  *Y_1_*: Adoption of/increased use of online grocery shopping due to the COVID-19 pandemic | *Survey question*: If you were to classify yourself, you would say that you are: (1) first-time on-line grocery shopper (i.e. started shopping for groceries online only as a result of COVID-19), (2) on-going online grocery shopping (i.e. shopped online for groceries before the pandemic), and (3) not an online grocery shopper (i.e. never buy groceries online)  *Survey question for on-going shoppers*: Overall, do you feel that your online grocery purchases increased as a result of the COVID-19 pandemic? (1) yes, (2) no, and (3) uncertain.  Y_1_ is a dummy variable equalling to 1 if a consumer is a first-time online grocery shopper and if an ongoing shopper and increased their online purchases; 0 otherwise. |
| *Model 2*  *Y_2_*: Tendency to continue the use of online channels for grocery shopping post-COVID | This model applies only to those consumers who, at the time of the survey, identified themselves as first-time online grocery shoppers and ongoing online shoppers.  *For first-time online shoppers*: Do you think you will continue buying groceries online even when the COVID-19 pandemic is over and all health measures/restrictions are removed? Y_2_ is a dummy variable that equals 1 if YES is selected, and 0 otherwise.  *For on-going online shoppers*: Do you think that your increased online purchases of groceries will continue into the future even when the pandemic is over? Y_2_ is a dummy variable that equals 1 if YES is selected, and 0 otherwise. |
|  |  |
| **EXPLANATORY VARIABLES** |  |
| **Perceived COVID threat** | |
|  | |
| Personality type in terms of response to COVID | *Survey question*: Which of the following groups best describes your attitude during the pandemic? |
| Indifferent | The **Indifferent**: “This is seriously being blown out of proportion; it’s just a flu. All these government rules and restrictions to confine the spread of the virus make no sense and only create inconveniences”. |
| Worrier | The **Worried/Concerned**: “I am very fearful of the future and I worry about my health a lot. I’m not willing to take any chances. News about new cases and deaths caused a fair amount of stress”. Dummy variable that equals 1 if this type and 0 otherwise. |
| Individualist | The **Individualist**: “I and my family will be fine. I’m more concerned about people acting irrational and engaging in unreasonable panic buying”. Dummy variable that equals 1 if this type and 0 otherwise. |
| Rationalist | The **Rationalist**: “I’m not concerned. All I can do is keep things and myself clean. I hope others do the same. News doesn’t worry me too much as I have this ‘keep calm and carry on’ mentality”. Dummy variable that equals 1 if this type and 0 otherwise. |
| Activist | The **Activist**: “I want to maintain social distance and wear masks not to just protect myself but to protect others as well. Protecting others is our social responsibility”. Dummy variable that equals 1 if this type and 0 otherwise. |
| Perceived susceptibility | *Survey question:* At the on-set of the COVID-19 pandemic (March 2020), how strong were your fears of catching the virus? The following response categories were offered: (1) I had no fears at all, (2) I hardly had any fears, (3) My fears were somewhat strong, and (4) I had very strong fears. Two dummy variables were created. Perceived susceptibility (high) is a dummy variable that equals 1 if the response option (4) was chosen and 0 otherwise. Perceived susceptibility (somewhat high) is a dummy variable that equals 1 if the response option (3) is chosen and 0 otherwise. |
| Perceived severity | *Survey question*: At the on-set of the COVID-19 pandemic (March 2020), how concerned were you about your health if you contracted the virus? The following response categories are provided: (3) I wasn’t concerned at all; I knew I’d be just fine even if I caught the virus, (2) I was somewhat concerned, and (3) I was very concerned. Perceived severity (high) is a dummy variable that equals 1 if option (4) is chosen and 0 otherwise; perceived severity (somewhat high) is a dummy variable that equals 1 if option (3) is chosen and 0 otherwise. |
|  |  |
| COVID_close contacts | *Survey question*: To the best of your knowledge, have any of your contacts (family members, relatives, colleagues at work, or friends) had serious complications from COVID-19? Dummy variable that equals 1 if YES. |
| Province | Province of residence is used as a proxy to capture the information about COVID-19 available to consumers. Maritime provinces (New Brunswick, Nova Scotia, and Prince Edward Island) and Newfoundland and Labrador serve as the benchmark category as they had the lowest rate of COVID-19 cases and the lowest rate of deaths due to COVID-19. Six dummies were created for Ontario, Quebec, Manitoba, Saskatchewan, Alberta, British Columbia. |
| Age | Continuous variable showing the age of the respondent. |
| Education | Categorical variable treated as continuous with the following ordered response categories: (1) Less than high school, (2) high school, (3) Apprenticeship or trades certificate or diploma; college or other non-university certificate or diploma, (4) university certificate or diploma, (5) university degree at BA level, and (6) post-graduate university degree. |
| Sex | Dummy variable that equals 1 if female and 0 otherwise. |
|  |  |
| **Socio-economic background** | |
| Children_ages 6-11 | Dummy variable that equals 1 if the respondent’s household had children between the ages 6 and 11 |
| Children_under 6 | Dummy variable that equals 1 if the respondent’s household had children under the age of 6. |
| Income | Annual household income; ordered categorical variable treated as continuous with the following response categories: (1) less than $20,000, (2) $20,000-39,999, (3) $40,000-59,999, (4) $60,000-79,999, (5) $80,000-99,999, and (6) $100,000+ |
| Change in income | The benchmark category is “no change in income”. A dummy variable that equals 1 if the respondent reported a significant or slight reduction in income and 0 otherwise. A dummy variable if the respondent reported an increase in income. |
|  |  |
| **Pre-COVID grocery shopping behaviour** | |
| Prior frequency of store visits | *Survey question*: Before the COVID-19 pandemic, how often did you do in-store grocery shopping? The following response options were provided: (6) Daily, (5) Every other day (2-3 times a week), (4) Once a week, (3) About once every 2 weeks, (2) About once a month, and (1) Less than once a month. The variable is treated as continuous variable with the higher value indicating higher frequency. |
| Shopping venue | A dummy variable that equals one if the respondent reported that they shopped at large supermarkets always or almost always prior to the pandemic. |
| Prior online | *Survey question:* Before COVID, according to your estimate, what percent of your total grocery purchases were done in-store versus online? |
| Grocery_importance | *Survey question:* Think about your average weekly meals before the COVID-19 pandemic. According to your estimates, what percent of weekly meals were coming from each of the following sources: (1) meals cooked at home from scratch, ingredients bought from grocery stores/supermarkets; (2) ready-to-eat items from grocery stores/supermarkets; (3) fully cooked meals from restaurants; (4) meals from fast-food places; (5) meals cooked at home from ready-to-go meal kits; (6) other, please specify. This variable is the sum of the percentages given for (1) and (2). |
|  |  |
| **Technological opportunities and abilities** | |
| Age | As discussed above |
| Education | As presented above |
| Income | As presented above |
|  |  |
| **Psychological predisposition to online purchases** | |
| Sex | Dummy variable that equals 1 if female and 0 otherwise. |
| **Past experience with online grocery shopping** | |
| Past experience | Dummy variable that equals 1 if the respondent indicated that their online grocery shopping experience has been positive and 0 if negative or neutral. |
| **Perceived advantages** | *Survey question*: To what extent do you agree with the following statements? The response categories are as follows: (1) strongly disagree, (2) somewhat disagree, (3) neither agree nor disagree, (4) somewhat agree, and (5) strongly agree. |
| Time savings | Online grocery shopping saves lots of time. |
| Economical | Online grocery shopping is more economical as it helps me stick to my grocery list and get good deals. |
| Greater product variety | Online grocery shopping offers a greater product variety than what’s available in stores. |
| Ease of use | Online grocery shopping is easier than in-store. |
| Pleasure | I view online grocery shopping as a pleasure rather than a chore. |
| Benefits | While I started using grocery shopping as a social distancing measure during COVID, I can now see lots of other benefits of shopping for groceries online. |
| **Perceived disadvantages** | *Survey question*: To what extent do you agree with the following statements? The response categories are as follows: (1) strongly disagree, (2) somewhat disagree, (3) neither agree nor disagree, (4) somewhat agree, and (5) strongly agree. |
| Over-spending | With online shopping it is much easier to lose control of how much is spent on food. |
| More costly | Online grocery shopping is more expensive due to additional costs and delivery. fees |
| Quality of fresh produce | Buying fresh produce online is a challenge, so I will always go to the store for perishable items in between my online orders. |
|  |  |
