# Supplementary material for: The demand for online grocery shopping: COVID-induced changes in grocery shopping behavior of Canadian consumers
Source: PLoS One. 2024 Feb 8;19(2):e0295538. doi: 10.1371/journal.pone.0295538 (PMC10852330; doi:10.1371/journal.pone.0295538)
Supplement: Attachment — Submitted filename: Response to Reviewers.docx [file pone.0295538.s003.docx]

**Responses to the reviewers**

First, we would like to thank the reviewers for their insightful comments as we believe that addressing these comments has significantly improved the quality of the paper. Here, we briefly outline the revisions made to address the reviewers’ concerns/suggestions; the full extent of the revisions is obvious from the resubmitted manuscript that highlights the changes made via the track changes word feature.

1. **Comment 1**: major concern.

*Response:* We have considerably expanded our knowledge of the existing literature and the set of references. We have added references to more than 60 articles [in addition to the references we had in the original submission], including references of the studies discussing the key theoretical models of consumer behaviour as well as more recent studies exploring the impact of the COVID-19 pandemic on consumer buying behaviour. As a result, we believe that the revised manuscript now has a much stronger review of the background literature and a much stronger theoretical base for the empirical analysis. While we do agree that the paper would highly benefit from a reconfiguration of the data analysis framework, more specifically, addressing the topic via SEM, unfortunately, the existing dataset (the existing collected survey data) is missing some key questions/respondents’ responses that would be required for validation and measurement of some of the key constructs. This weakness of the existing dataset makes application of SEM technique impossible. Given the existing data we can only use one equation econometrics (Logit) estimation technique. We did add a mention of this weakness in our conclusions and one of the directions for our future research can be an extension of this study that would involve a new survey design and new data collection to ensure that we can explore and validate via SEM the multiple models of consumer behaviour that have been referenced in this work to set up the foundation for the Logit regression model.

2. **Comment 2**: the idea of “changes” and how this induces the idea of longitudinal research.

*Response:* While the survey was administered at one point in time, in January 2022, meaning that we did not collect longitudinal data, the survey was administered almost 2 years after the onset of the pandemic. At the time when the survey was being administered, COVID was still a big issue (Canada was starting a new Omicron wave with the confirmed case numbers increasing rapidly); some of the government imposed restrictions (e.g. social distancing, mandatory masking, etc.) and policies were still in place. The survey questions explicitly asked participants to describe their grocery shopping behaviour prior to the pandemic, at the onset of the pandemic, and “currently” [i.e. January 2022]. Since the survey was not run too early into the pandemic (e.g. summer 2020), it is believed that in January 2022 consumers were fully aware of their “complete” adaptation to the pandemic and all the changes in buying behaviour the consumers had to make. So, although the data were not collected in a longitudinal manner, given the timing of the survey and the nature of the survey questions we were able to explore the COVID-induced “changes”. In the “Survey design” section we added a brief paragraph highlighting the fact that the questions were set up in such a way that consumers could report the changes in their grocery buying habits at different points in time.

3. **Comment 3**: Introduction

*Response:* We have revised Introduction to clearly articulate the aim, the importance, and the research contribution of the study. While many research papers briefly mention the data and the findings in the introduction, we agreed with the first reviewer’s suggestion and removed the paragraph that briefly mentioned the survey data and the findings. Instead, we focused on highlighting the contribution of the paper to the existing research.

4. **Comment 4**: Background literature and theoretical framework

*Response:* While in the original submission we tried to keep everything concise, having seen the first reviewer’s comment we were in full agreement that in many instances we presented the information in a very brief way. We have now created two separate sections. Section 2 presents Background literature, where we provide a brief review of the literature on consumer behaviour during pandemics in general and during the COVID pandemic in particular. This section now includes many recent studies on adaptations in consumer behaviour during COVID and provides a smooth link to a switch to online grocery shopping – the research interest of the paper – as one aspect of these adaptations.

Section 3 uses the existing literature to build a theoretical model that focuses on consumer adoption of online grocery shopping; in this section we now clearly articulate the hypotheses (propositions). We have tried to provide theoretical perspectives and references to any claims (ideas) that are discussed.

Note that we scraped the previous “Background literature and theoretical framework” and have completely re-wrote this section that is now Section 2 and Section 3 of the revised manuscript. We do agree that this section was previously under-referenced and the results of more than 60 additional studies have been added to support the discussion in Section 2 and 3.

5. **Comment 5**: Research methodology.

*Responses:* Most of the questions in our survey questionnaire intended to elicit the changes that consumers had to make in response to the restrictions posed by the pandemic and to see which of these changes will have a long-lasting life beyond COVID. Since we had a slightly different goal in mind, we did not set up the questionnaire to include the scale items for multiple constructs to conduct a SEM analysis using the data. This is the weakness of this dataset and the reason why SEM technique can’t be applied to these data as mentioned above.

6. **Comment 6**: Results

*Response:* Since most of the variables in our dataset are either dichotomous or ordered categorical, we agree with the first reviewer that the use of Probit cannot be justified as the residuals are likely not to be normally distributed. We have re-estimated the regression model using Logit, which is not built upon the assumption of normal distribution of the error term. The estimated (updated, Logit based) coefficients are similar (in terms of direction of the impact and its significance) to what we had before using Probit. We made the changes to our discussion of the results to reflect the new estimates and the significance of the impacts. We also expanded our discussion on certain aspects of adoption/expansion of online grocery shopping.

We hope that now that we have completely re-written the theoretical model section and explicitly stated the propositions (hypotheses), the reviewer will find that the discussion in the results section aligns much better with the theoretical model.

7. **Comment 7**: Conclusions and policy implications

*Response:* We have made the appropriate adjustments to the concluding section of the paper to address the reviewer’s comments.

8. **Comment 8:** Suggestion to have English editing on the paper

*Response:* The paper has been professionally edited (proofread by a professional editor specializing in Economics) before resubmission to ensure proper use of language throughout the paper.

Summarizing, we would like to thank the reviewers for their time providing such detailed and valuable comments. We believe that, in the current revised version of the manuscript, we have addressed the reviewers’ comments fully and hope that the reviewers will find the revised manuscript to be of a much better quality, worth of publication in such a high impact journal as PLOS ONE (Economics).

Sincerely,

Viktoriya Galushko
