# Supplementary material for: The demand for online grocery shopping: COVID-induced changes in grocery shopping behavior of Canadian consumers
Source: PLoS One. 2024 Feb 8;19(2):e0295538. doi: 10.1371/journal.pone.0295538 (PMC10852330; doi:10.1371/journal.pone.0295538)
Supplement: Attachment — Submitted filename: Response to Reviewers.docx [file pone.0295538.s004.docx]

**Responses to the reviewers**

First, we would like to thank the reviewers for their insightful comments as we believe that addressing these comments has significantly improved the quality of the paper. Here, we briefly outline the revisions made to address the reviewers’ concerns/suggestions; the full extent of the revisions is obvious from the resubmitted manuscript that highlights the changes made via the track changes word feature.

1. We have substantially expanded the Results section to provide a better link between the section developing the theoretical model and the sections explaining the results. We have improved the statistical analysis of the data as well as have provided information on a profile of respondents, by key consumer characteristics.
2. We added the suggested references to better link the findings from this Canadian study to the findings from some other recent studies. Unfortunately, the literature on online grocery shopping behavior in the Canadian context is very scarce so we could not expand discussion in this front.
3. We’ve also added references to some studies that used a similar approach, more specifically, logistic regression analysis, to analyze consumer adoption of e-commerce.

Summarizing, we would like to thank the reviewers for their time providing such detailed and valuable comments. We believe that, in the current revised version of the manuscript, we have addressed the reviewers’ comments fully and hope that the reviewers will find the revised manuscript to be of a better quality, worth of publication in such a high impact journal as PLOS ONE (Economics).

Sincerely,

Viktoriya Galushko
